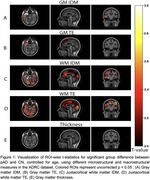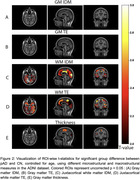# Diffuse juxtacortical white matter texture changes in pre‐clinical Alzheimer’s disease

**DOI:** 10.1002/alz.094122

**Published:** 2025-01-09

**Authors:** ADEMOLA E ILESANMI, Xueying Lyu, Emily McGrew, Paul A. Yushkevich, Sandhitsu R. Das, David A Wolk

**Affiliations:** ^1^ University of Pennsylvania, Philadelphia, PA USA; ^2^ Perelman School of Medicine, University of Pennsylvania, Philadelphia, PA USA

## Abstract

**Background:**

Detection of neurodegeneration during preclinical stages of Alzheimer’s disease (AD) is critical for disease prediction and monitoring. Macrostructural brain changes (volume/thickness) during preclinical AD are often not detectable in cross‐sectional studies. Microstructural changes manifested as alterations in texture features may capture subtle neurodegeneration at this stage. Here we investigated the discriminative ability of texture‐based radiomics features in both gray and juxtacortical white matter in preclinical AD.

**Method:**

The dataset included amyloid negative (CN) and amyloid positive (preclinical AD, pAD) cognitively normal individuals from two independent cohorts (ADRC: 122 CN, 35 pAD; ADNI: 183 CN, 95 pAD). T1‐weighted MRI was segmented. Subsequently, we extracted thirty texture features each from 100 gray matter (GM) regions of interest (ROI) and corresponding adjacent white matter (WM) ROIs extending 2mm from the GM/WM interface. Principal Component Analysis was employed to reduce these features to eight: total energy (TE), sum variance, skewness, sum average, entropy, contrast, kurtosis, and inverse difference moment (IDM).

**Result:**

Initial analysis in the ADRC cohort demonstrated group differences in TE and IDM in juxtacortical white matter regions broadly throughout the brain (Figure 1C, D), but not in the grey matter (Figure 1A, B). Given the exploratory nature of the analysis in the ADRC, we then examined TE and IDM in ADNI in groups defined in the same manner. These findings were largely replicated in the ADNI dataset (Figure 2). Conversely, gray matter thickness measures in both cohorts displayed minimal group discrimination (Figures 1E and 2E).

**Conclusion:**

Texture features in juxtacortical white matter were sensitive to pAD in two independent cohorts, whereas GM thickness, a macrostructural measure, was not. These data suggest that texture features, a possible surrogate of microstructural changes, may be particularly sensitive to early downstream effects of AD. However, the diffuse nature of these findings across the brain suggests the possibility that they are related to the presence of cerebral amyloid rather than the more focal effects that would be expected with tau‐related neurodegeneration. Future work will test the longitudinal nature of these texture findings and their relationship to prediction of future cognitive decline.